# Three-Dimensional Radiographic Outcome of Free-Handed Flaplessly Placed Mini Dental Implants in Edentulous Maxillae after 2-Years Function

**DOI:** 10.3390/jcm9072120

**Published:** 2020-07-05

**Authors:** Luc Van Doorne, Pedram Gholami, Jan D’haese, Geert Hommez, Gert Meijer, Hugo De Bruyn

**Affiliations:** 1 Department of Oral and Maxillo-Facial Surgery, Medical & Cosmetic Treatments, Cosmipolis Clinic, Sint Claradreef 77, 8000 Brugge, Belgium; 2 Department of Periodontology and oral implantology, Oral and Maxillo-Facial Surgery, Dental Radiology, Ghent University Hospital, Corneel Heymanslaan 10, 9000 Ghent, Belgium; drpedramgh@hotmail.com (P.G.); Geert.hommez@uzgent.be (G.H.); or Hugo.deBruyn@radboudumc.nl (H.D.B.); 3 Faculty of Medicine and Health Sciences, Ghent University, St Pietersnieuwstraat 33, 9000 Ghent, Belgium; 4 Department Periodontology and Oral Implantology, Department of Maxillo-Facial Surgery, Institute of Health Sciences, Radboud University Medical Centre, 6525 GA Nijmegen, The Netherlands; Jan.Dhaese@radboudumc.nl (J.D.); Gert.Meijer@radboudumc.nl (G.M.)

**Keywords:** free-handed, flapless surgery, mini dental implants, CBCT evaluation, surgical complications, maxillary overdenture

## Abstract

Background: Free-handed, flaplessly placed mini dental implants (MDIs) are a valuable, more affordable and minimally invasive treatment to support overdentures in fully edentulous jaws, especially for medically compromised patients. However, critical 3D radiographic evaluation is lacking. This multicenter prospective case series assessed clinical outcome and carried out 3D- cone-beam computerized tomography (CBCT) analysis of free-handed flaplessly placed one-piece maxillary MDIs by an experienced maxillofacial surgeon. Methods: Thirty-one patients suffering from an ill-fitting maxillary denture relating to compromised bone volume (as confirmed on CBCT), with a dentate mandible, were selected. They received 5–6 MDIs free-hand flaplessly placed and mentally guided with preoperative CBCT. Final connection and attachment activation took place six months later. After two years each implant was individually assessed with CBCT for perforations on eight sites. Implant survival, prosthetic failure, clinical stability and sinus/nasal complaints were registered after three years. Results: 32/185 (17.3%) MDIs failed during the provisional loading with non-activated attachments; 17 replacements in 10 patients were performed. Of the 170 actively loaded 170 MDIs, 82.3% survived and 27/31 prostheses (87%) were fully functional. In total 98/170 MDIs showed no perforation. Based on 1360 CBCT observations, 231 perforations (16.9%) were registered. Of most perforations 37 (25%) were observed at the apical tip and 37 were positioned (21%) into the sinus/nasal cavity, although without clinical complaints. Conclusions: Given the compromised population, the minimally invasive procedure and the low treatment cost involved, a failure rate of 17% is substantial, however clinically acceptable given the critical bone condition. However, even in experienced surgical hands, freehanded and flapless placement yield a high risk for implant perforation, although this did not necessarily lead to complications.

## 1. Introduction

Flaplessly placed mini dental implants (MDI) can serve as less painful and affordable [1] implant treatment to support overdentures for the rehabilitation of atrophic edentulous jaws. Fear of postoperative pain, complications [2,3] and elevated costs [4], especially in cases where bone reconstruction is necessary, are the most common barriers for implant treatment in edentulous elderly and especially in medically compromised or low-income populations. MDIs have been used quite often to retain mandibular overdentures. Jawad et al. [5] reported a total failure of 4.4% based on a systematic review summarizing the outcome in 475 patients derived from 17 clinical studies. Prospective studies indicate that scarce and inconsistent information is available for MDIs placed in the maxilla. Lemos et al. [6] reported a failure of 31.7% based on only three clinical studies. These studies presented a failure of 32.1% at one year follow-up [7], 22.2% after three years [8] and 33.3% after two years [9] respectively, which is much higher than the 4.9% derived from 15 studies in the mandible. A retrospective study [10,11] investigated 5640 MDIs over a period of 12 years with different prosthetic solutions including fixed, partial or full removable dentures. Overall, survival for the MDIs was 93.1% in the mandible and 91.3% in the maxilla, but 14.8% of MDIs failed when retaining maxillary overdentures after three years.

Flapless surgery uses rotary burs or a tissue punch to gain access to the bone without flap elevation, so the periosteal vascular supply and surrounding soft tissue are not disturbed. This minimally invasive surgical technique has many advantages [12]. It allows a shorter surgical time with minor postoperative discomfort. It can preserve hard and soft tissues around the implants which accelerates the healing process [13]. Flapless surgery can be performed either with 3D planned surgical guides, with computer-assisted navigation [14,15] or free-handed so-called mentally guided, albeit it is recommended only for experienced surgeons. Voulgarakis et al. [16] reported no differences for the three different techniques on survival rate, marginal bone loss and complications. Guided flapless surgery seems to be more accurate for ideal implant positioning [17]. However, increased efforts using 3D implant software, preoperative planning time, accuracy issues in transferring the implant planning to the surgical field and higher technical costs involved, are important drawbacks [16,18]. Free-handed flapless surgery is a more accessible implant treatment procedure although surgical experience with the technique is mandatory, and a correct estimation of the alveolar bone morphology during the drilling procedure is a prerequisite. To date, little is known of the exact implant position when free-handed flapless surgery is performed [16]. This may increase the risk for perforations, dehiscence or fenestration resulting in surgical complications or compromised implant integration [16]. Besides the risk of lingual or palatal perforation of the cortical plate, perforation of the sinus or nasal floor could lead to sinus infection [19] or rhino-sinusitis [20].

The aim of this study was to critically scrutinize the outcome of free-handed flapless placement of MDIs in edentulous maxillae, using three-dimensional assessment on cone-beam computerized tomography (CBCT) CBCT, two years after placement by an experienced surgeon.

## 2. Materials and Methods

### 2.1. Patient Inclusion

The clinical study was designed as a multicenter prospective cohort study. Subjects aged 50 years or older were included. They were referred because they were dissatisfied with their conventional full maxillary denture. Patients were either partially or fully dentate in the mandible with natural dentition, an implant-supported fixed or partial removable prosthesis. Patients with uncontrolled systemic diseases, immunocompromised conditions, previous treatment with oral or intravenous bisphosphonates and a history of alveolar bone reconstruction or radiotherapy in the maxillofacial area were excluded. Pregnancy or under-age patients were not applicable as exclusion criteria given the 50+ age population in this study. Patients were treated in a 17 months period from August 2015 until January 2017. All included patients provided an informed consent before treatment and the study protocol was approved by the Ethical Committees of the General Hospital AZ ZENO Knokke-Blankenberge and the Ghent University Hospital. (Belgium, registration number B670201422937).

### 2.2. Mini Dental Implants

**Figure 1:**
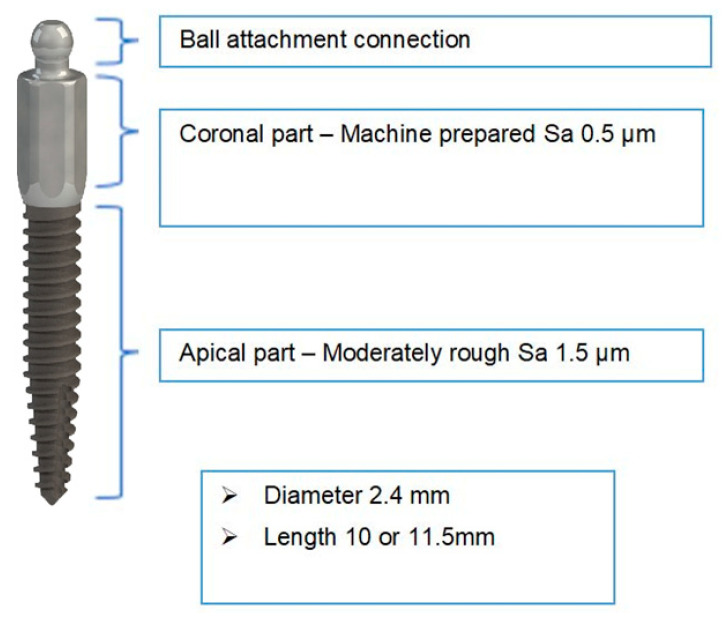
ILZ prototype mini-implant Southern Impl., Inc., Irene, South Africa.

The MDIs (ILZ, Southern Impl., Inc., Irene, South Africa) (Figure 1) were designed as one-piece implants with high strength and made of pure titanium Class 4. The diameter was 2.4 mm coronally and the threaded part was 10 or 11.5 mm long and with a surface roughness (Sa) value of 1.5 μm. The transmucosal part was machined with a Sa value of 0.4 μm, 4.8 mm long and had a coronal ball abutment of 1.8-mm diameter on the top.

### 2.3. Treatment Procedure

**Figure 2:**
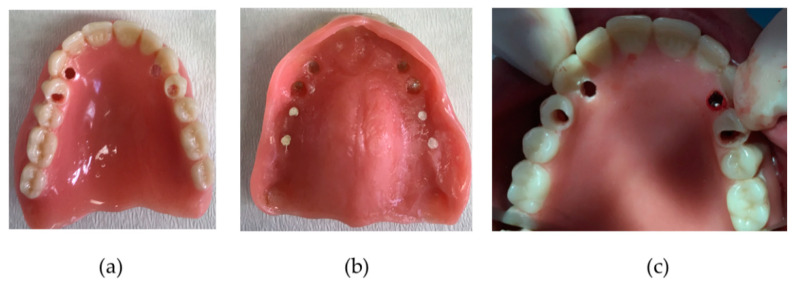
Provisional denture as a surgical guide. (**a**) palatal view; (**b**) mucosal view; (**c**) intra-operative view with direction pin visible in the prepared burr hole region 2.3.

Prior to surgery, the existing conventional maxillary denture was relined or renewed to ensure proper adaptation to the soft tissues. Radiopaque gutta percha markers were installed in the base of the denture at the incisor, premolar and molar location before the preoperative CBCT was taken with Planmeca Promax 3D-dental CBCT and analyzed with Planmeca Romexis^®^ software (Planmeca Oy, Helsinki, Finland). The examination was performed with the patient in a stabilized sitting position. First a scout image was performed to scrutinize acceptable horizontal maxillary jaw positioning before final DICOM image execution. The specific imaging protocol used for the maxilla was a voxel size of 200 μm, a field of view of 80 mm × 50 mm, 90 KV, a variable range of 5–8 mA depending of patient morphology/gender and with an exposure time of 12 s, thereby reducing patient’s dose to the minimum level, according to the “as low as reasonably achievable” (ALARA) principle [21]. By reverse calculation, the preferred implant locations were planned by estimation. Surgery was performed by one surgeon (LVD) under local anesthesia. Placement of six MDIs was planned using a free-handed flapless approach. Before surgery, the gutta percha markers were removed and the aimed implant position was transferred through the prepared holes by perforating the gingiva and the alveolar cortical bone. The denture was then removed and with bi-digital palpation of the alveolar process, further preparation of the implant site was accomplished with a 2.0-mm-diameter final burr (Figure 2).

**Figure 3:**
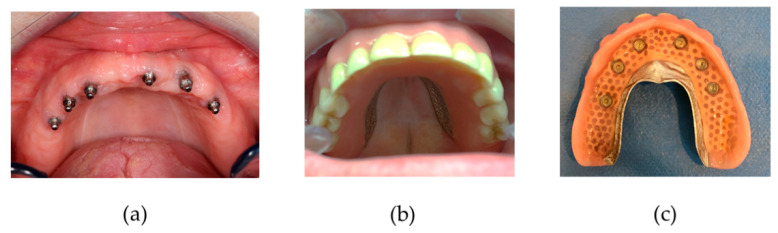
(**a**) Maxillary mini dental implant (MDI) position; (**b**) final denture in situ; (**c**) final denture palatal side with retention caps and o-rings.

Whenever bone perforation was assessed during drilling or registered during palpation, the direction of the burr was slightly changed in an attempt to stay as parallel as possible with the other positioned implants. Implants were inserted manually, and care was taken to position the coronal ball about 2 mm above the gingiva. The patients were advised to refrain from wearing their denture for one week postoperatively. At the one-week postoperative check-up, the denture was adapted with retentive soft relining material (Coesoft, GC America, Chicago, Illinois, US). Patients received oral hygiene instructions with a soft toothbrush and were followed up on a regular basis by either the surgeon or prosthodontist for relining whenever required. The final prosthetic connection was established at least 6 months following implant placement (Figure 3). Figure 3 shows the new metal-reinforced, horse-shoe denture with activated attachment which clip onto the ball parts of the MDI.

Prosthetic failure was defined as the inability of the available MDIs to support a horse-shoe overdenture with adequate retention. Some implant failures were acceptable as long as retention was not jeopardized. Where necessary, the failed implants were replaced.

### 2.4. Clinical Evaluation

During the 2-year control visit, MDI survival as well as clinical symptoms and sinus/nasal complaints were assessed with the rhinosinusitis symptom severity score (RS-SSS) (Supplementary Materials) [22].

### 2.5. Radiological Evaluation

**Figure 4:**
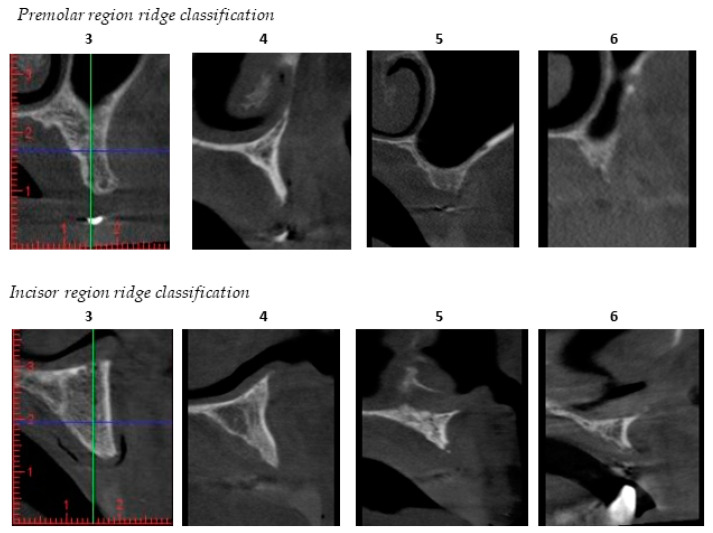
Cawood–Howell classification [23] of the maxillary alveolar ridge applied to cone-beam computerized tomography (CBCT) images in the patient population studied. This classification describes a progressive bone resorption pattern in 7 scores; score 1 being where a tooth is still present; and score 7 being extensive alveolar bone resorption. Score **3** and **4** represents bone which is sufficient in height, but too narrow in the crestal and midfacial zone (knife edge). Scores **5** to **6** show insufficient bone height and/or width to allow conventional implant placement without additional grafting procedures. Two examiners, the treating surgeon (L.V.D.) and the oral radiologist (G.H.), assessed all CBCTs independently to describe the quantity of available bone.

Maxillary bone volume was examined with axial sections of the pre-operative cone-beam computerized tomography (CBCT) in the preferred premolar/canine and incisor regions and categorized according to Cawood and Howell classification [23] as shown in Figure 4.

**Figure 5:**
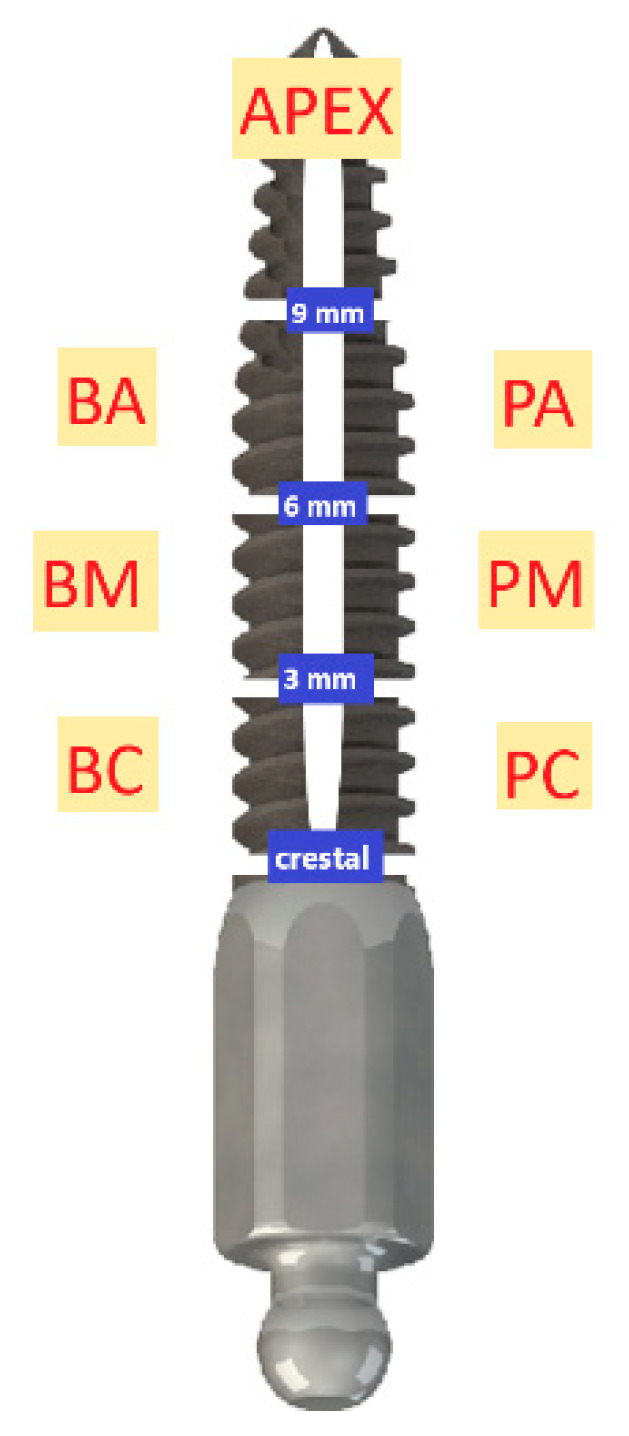
Different MDI sections at 1 mm (crestal), 3 mm, 6 mm and 9 mm with defined MDI bone-to-implant contact regions: BC: buccal coronal, PC: palatal coronal, BM: buccal middle, PM: palatal middle, BA: buccal apical, PA: palatal apical, apex or apical

A CBCT was taken 2 years after implantation. Bone width at different sections of the MDIs, as indicated in Figure 5, were calculated: 1 mm below the margin of the coronal machined part and the apical rough treated part (crest) and, respectively 3, 6 and 9 mm below the crest.

**Figure 6:**
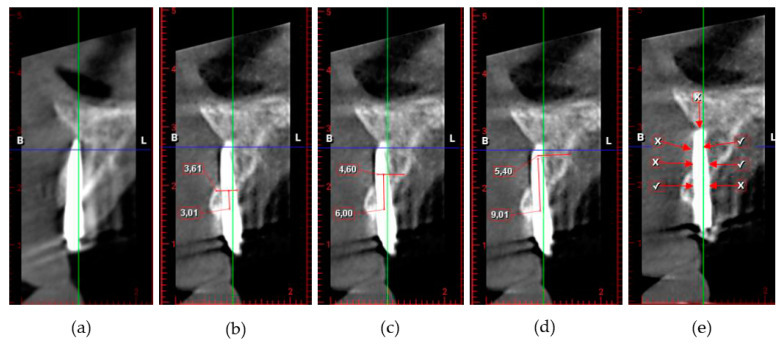
(**a**) CBCT cross sectional view of MDI placed in location 23; (**b**) measurement of bone thickness at 3 mm; (**c**) measurement of bone thickness at 6 mm; (**d**) measurement of bone thickness at 9 mm; (**e**) perforations marked with (X) and bone-to-implant contact (BIC) buccal/palatal marked with (V).

The evaluation procedures are demonstrated in Figure 6 on a sectional view region 2.3.

Additionally, the degree of swelling of the sinus mucosa (Schneiderian membrane), the width of residual bone above the MDI apex upward to the sinus/nasal cortex, the axial measurement of bone-to-implant contact (BIC) and the number of perforations were assessed. Perforations were registered at the buccal, palatal, sinus/nasal cortex, the nasopalatine foramina and the MDI apex.

### 2.6. Statistical Analysis

This study is an exploration of the results of an approach for treating challenging patients. Individual implants were clustered within patients, which leads to dependency between observations and usually has consequences for the statistical analysis. Those dependencies result in invalid estimates of variance, a problem that may be addressed with multilevel models or survival analysis using shared frailties. Due to the strong descriptive character of our study, statements related to variances or derived quantities (such as *p*-values) were not presented and only means, percentages and survival levels are provided. For intra-and inter-rater reliability, obtained after repeating the measurements independently, Cohen’s weighted kappa coefficient was calculated. Kaplan Meijer analysis was performed including the original and the replaced (failed) implants. Statistical analysis was performed using SPSS version 25 (IBM SPSS, statistics for Windows, version 25.0, Business Analytics, Amonk, New York, NY, United States).

## 3. Results

### 3.1. Patients

Thirty-eight patients were informed about the study initially. Thirty-one out of the 38 patients finally signed the informed consent and were enrolled; 14 (45.2%) females, 17 (54.8%) males with a mean age of 62.30 (SD 9.28). Twenty-one (67.7%) patients had a natural antagonistic dentition, 5 (16.1%) a combination of partial denture and natural teeth and 5 (16.1%) had an implant overdenture. The pre-operative relined prosthesis was adapted and early installed on 6 non-splinted MDIs. Full activation with a horse-shoe denture was executed on average 0.8 years (SD 0.29) after implant placement. The last examination including the CBCT control was 2.9 years (SD 0.88) after implant installation.

A total of 185 MDIs were placed initially. Healing was uneventful in 15 patients (48.4%) with 89 implants (in 1 patient 5 MDI’s were installed). In 16 patients (51.6%) 32 MDI’s were lost during provisional loading; 8 patients lost 1 and 4 lost 2 MDI, 2 patients lost 3 MDI and 2 lost 5 MDI resulting in an initial MDI failure of 32/185 (17.3%). Lost implants were replaced in 10 patients, with 17 new MDIs. The major MDI failures occurred during the initial healing phase. Kaplan-Meier life table estimated a survival of 86.3% at six months, 84% at one year and 82.3% at two years. Additionally, after 3 years one patient encountered 2 MDI fractures (with 3 months interval). During clinical assessment 2 patients reported some moderate rhino-sinusitis complaints with RS-SSS.

In the 2 patients with 5 MDI losses, 1 patient received a prosthetic rehabilitation with 6 two-piece Narrow Diameter Implants (NDI) of 3-mm diameter. The other patient refused further implant treatment and preferred to continue with a new removable denture fixed with adhesive glue. This is also the case for the patient with the fractured MDIs. In 1 patient, a heavy smoker, we replaced 2 initial MDI ILZ implants with 2 two-piece NDI and a new overdenture. Hence prosthetic failure was encountered in 4/31 patients, yielding a prosthetic success rate of 87% after 3 years.

### 3.2. Radiological Evaluation

**Figure 7:**
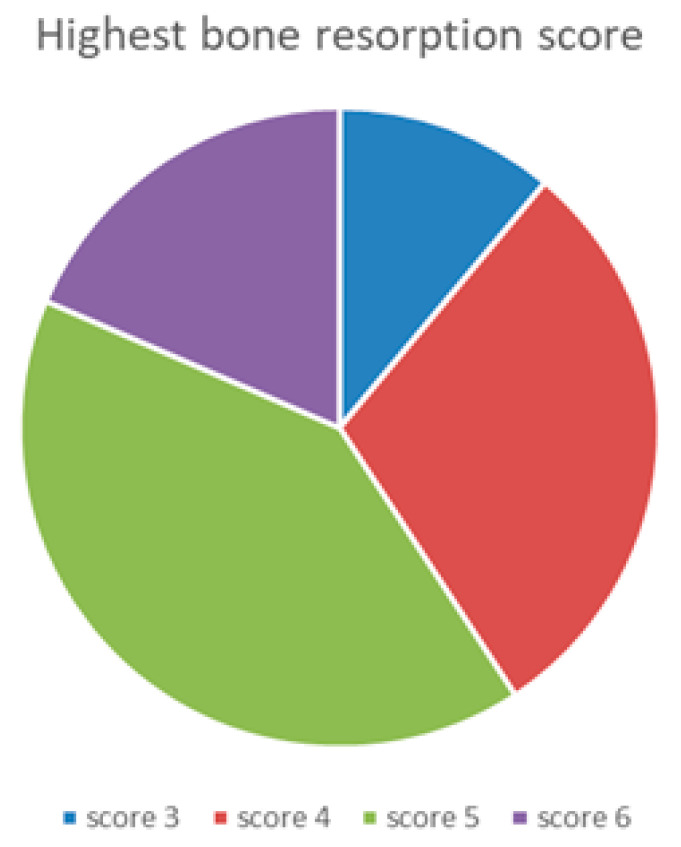
Highest bone resorption scores registered per patient according to Cawood and Howell [23].

Initial Cawood and Howell CBCT evaluation demonstrated the maxillary resorption of the included patient population. The Cohen’s weighted kappa for inter-examiner reliability for assessed resorption pattern was 0.739 in the premolar-canine and 0.837 in the incisor region, which is considered ‘substantial’ and ‘almost perfect’, respectively. Nearly 40% of the individual implant sites had a resorption score of 3–4. The highest score per patient was 3–4 in 41% and 5–6 in 59% of the subjects, pointing out that regular size implants were no option (Figure 7).

In total 170 MDIs were examined at the 2-year control visit. The CBCT control was carried out on average 2.88 (SD 0.88) years after surgery. The mean bone width is depicted in Table 1 and varied between 5.63 mm at the crest up to 6.61 mm apically. This confirms that the average bone width at the top of the crest, 3 mm more apically and 6 mm more apically, is too narrow for regular diameter implants.

**Figure 8:**
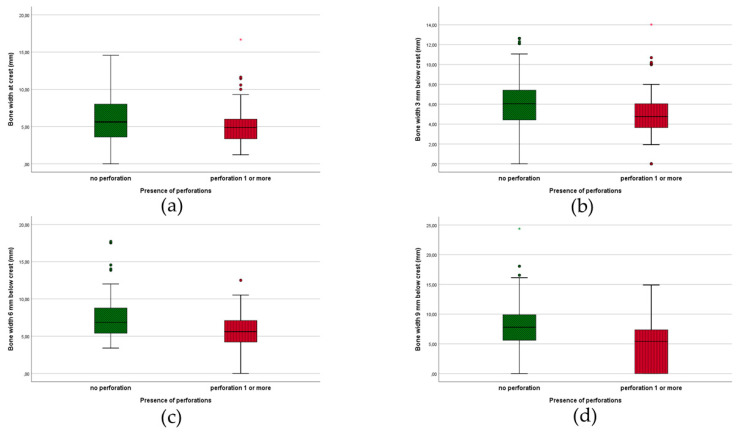
Presence of perforations in relation to bone width at different MDI levels (**a**) crestal; (**b**) 3 mm; (**c**) 6 mm; (**d**) 9 mm.

The mean apical level or distance from the implant apex to the cortical bone base of the maxillary sinus or nose was 0.40 mm (1.24 mm SD; 9.43 mm Range). Evaluation of the mean thickness of the Schneiderian maxillary sinus membrane was 0.64 mm (2.02 mm SD; 19.8 mm range). The mean BIC measured buccally 0.43 mm (1.43 mm SD; 10.2 mm range) and palatally 0.36 mm (1.12 mm SD; 6.08 mm range). Based on 37 sinus/nasal perforations, the mean length of perforation through the bone was 0.57 mm (1.38 mm SD; 5.86 mm Range). Figure 8 demonstrates the presence of 1 or more perforations with respect to the measured bone width at different intersections of the alveolar crest.

The occurrence of perforations is summarized in Table 2.

**Figure 9:**
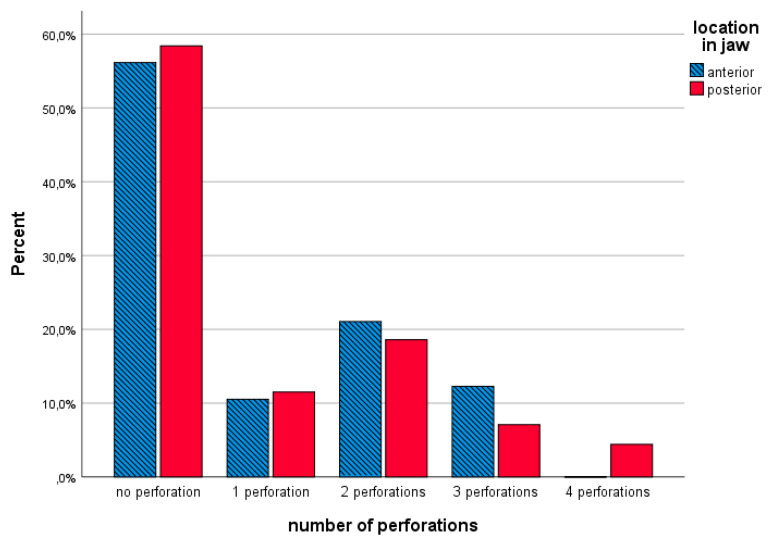
Perforation percentage in the anterior and the posterior jaw location.

Of a total of 1360 observations, 231 perforations (16.9%) were recorded. Apical tip 43 (25%) and sinus/nasal 37 (22%) perforations accounted for the major part. In terms of jaw location (anterior, posterior) perforation percentages are depicted in Figure 9.

## 4. Discussion

To the best of our knowledge this is the first clinical study scrutinizing the positioning of such an extensive number of maxillary MDIs placed free-hand, flaplessly, by means of CBCT evaluation. Klein et al. [24] and the ITI consensus statement of 2018 [25] classified implants with a diameter of 3.5 mm or smaller as Narrow-Diameter Implants and classified them in three subcategories from 3.3 mm to 3.5 mm, 2.5 mm to < 3.3 mm and implants with a diameter of <2.5 mm; these were defined as “Mini-implants”. Reducing the diameter of the implants increases the risk of fractures due to lower mechanical durability [26,27]. Fatigue fracture may occur in MDIs after a long period of function [27]. Fatigue can be considered a progressive process in which first cracks are being generated and then grow steadily to implant fracture [28]. Furthermore, the MDIs used in the present study were very narrow and the intraoral component did not allow splinting of the implants. This may have increased the risk of implant failure. Future hardware modifications can overcome this technical drawback.

In our study, the estimated failure rate for maxillary MDIs was 17.7% after two years, which is lower than the 31.7% failure evaluated in a systematic review [6]. From 185 MDIs placed initially, 32 were lost and 17 sites required replacement (when indispensable for a safe prosthetic loading in the long run). It would be unethical not to do so because too few remaining implants would survive the heavy load from the horseshoe denture. This is especially true given the critical bone conditions. As such, the presented outcome is an analysis of the entire treatment philosophy, including original and renewed MDIs. Obviously, implant loss is an unfavorable outcome. Despite that, nearly half of all treated patients experienced an implant failure. Regarding prostheses; 87.10% of the prostheses were fully functional prior to as well as after the retreatment. In maxillary implant-retained overdentures using four to six regular implants, the 5-year survival rate varies between 97% and 100%. This includes different prosthetic retention systems [29], different locations in the jaw (anterior, posterior) with or without sinus lift [30,31] and with or without opposed (partial) natural dentition [32]. However, very often, only the happy few can undergo these heavier procedures, thus supporting the suggestion that the MDIs may offer feasible alternatives to enhance Oral Health Related Quality of Life that outweighs the risk of implant loss.

Implementation of new radiological CBCT detection techniques allows the monitoring of crack development and evolution of small cracks in implants. This is known as “structural health monitoring” [28]. In the current study, fracture was encountered in only one patient with two implant fractures (with three months interval) after three years. Hence no microcracks were observed in these implants in our 2-year CBCT investigation. Mechanical complications, as implant fracture, are categorized as late implant losses and can be divided in mechanical damage of the implant, its components or the suprastructure supported by the implants [33]. The latter two imply higher costs for the patients over time. One piece MDI implants placed in adequate numbers are hence favorable from this perspective. No expensive adaptations are necessary to preserve patient’s denture comfort. Fractured MDIs can even be preserved when subgingival fracture is apparent. Moreover, MDIs can be replaced atraumatically with the presented free-handed flapless technique. A review [34] assessed the survival rate of 1.8 mm up to 4.1 mm wide implants placed in both arches. An overall survival of 90% was calculated based on 32 studies, however the survival was noticeably lower in the implants with smallest diameter. In eight studies even a 100% survival was noted. In another multi-center study [35] more than 700 MDIs were placed in 133 patients yielding a survival of 94.3% in the maxilla and 95.7% in mandible. They reported four fractures out of 402 mandibular MDIs, but none out of the maxillary ones. In a prospective five-year study [36], patient satisfaction with MDIs retaining mandibular overdentures was investigated and a significant increase in patient satisfaction in terms of eating hard and soft food, talking, appearance, comfort, healing process and socializing was reported. Reflecting on the available literature [34], it seems that implants with a standard implant diameter (SDIs) show higher survival rates than MIDs with a diameter below 3.0 mm. It also appears that MDIs placed in the mandibular arch have a higher and better survival rate compared to MDIs in the maxilla. In our study, most failures occurred during the initial healing phase suggesting either absence of osseointegration or loss of attained osseointegration due to overloading. However, care was taken to create space between the supracrestal implant part and the relined denture base, unwanted and premature loading cannot be excluded because the masticatory load with the ill-fitting denture was unaccounted for. Numerous factors may influence the survival of an implant comprising, among others, the amount of bone surrounding the implant and the quality of that bone as well as stress distribution to the surrounding bone [37]. In our study, we critically analyzed the surrounding bone with CBCT around the MDIs. Evaluation of the alveolar crest width at different sagittal levels reflects the classical maxillary edentulous morphology described by Cawood and Howell [23]. It is generally accepted that at least five- to six-millimeter-crestal width is advised for the placement of narrow diameter (3 mm) or regular diameter (4 mm) implants. As pointed out in Table 1, the average bone width at various levels show that a majority of the sites are too narrow for the placement of regular implants. In the treated population, placement of implants of three to four millimeters would result in implants being incompletely embedded in the bone. On individual site level, only 44.1% and 31.2% of the sites were sufficiently wide to allow placement of regular three- or four-millimeter-diameter implants. However, when considering that ideally six implants are required per patient, in order to retain a horseshoe dental prosthesis, only in four subjects was it feasible to install six implants of three-millimeter diameter, and only in two subjects was it feasible to install six implants of four millimeters. However, none of the selected patients could receive those regular diameter implants in preferable positions vis-a-vis proper distribution over the jaw, an acceptable inter-implant degree or proper location aiming for axial implant loading. This points out the compromised conditions tested in this clinical trial whereby other, more conventional treatment approaches were not feasible. MDIs were the only solution in order to avoid extensive grafting.

In case of a smaller alveolar width, placing MDIs in a free-handed and flapless manner, results in a higher perforation risk, as visualized in Figure 6 and Figure 9. At every measured level, a medium alveolar width of 6–7 mm seems to be safer, while 5–6 mm is more liable to perforate with 2.4-mm diameter MDIs. The smaller the alveolar process, the higher the risk of perforation. This seems logical but could serve as a clinical guideline when limited surgical experience with free-handed flapless surgery is available. Van de Velde et al. [38] described in their ‘in vitro’ model study a significant risk of perforations and dehiscences when four-millimeter-diameter implants were used in a freehanded and flapless manner. The outcome was not influenced by the level of experience with implant surgery. They concluded that correct implant placement with freehanded flapless surgery is difficult to perform and suggested computer aided surgical guides. Undoubtedly the introduction of three dimensional CBCT implant planning software, computer-aided surgery with pre-planned surgical guides or dynamic/static computer assisted navigation procedures are important achievements in optimizing 3D implant-positioning [39]. Although guides have been suggested for flapless implant placement to increase the accuracy and to improve the clinical outcome in the edentulous maxilla [40], contradictory results are published. D’Haese and coworkers [41] reported an apical deviation of up to 4.2 mm deviation. A systematic review [18] critically reports on 3D-guided surgical methods in the context of increased radiation dose (CT or CBCT), costs (planning software, surgical guides, CBCT), effort (familiarity with 3D-implant software), time (preoperative planning) and accuracy of the transfer of implant planning to the surgical field. Inclusion of more technologically-advanced techniques yields a price that increases the financial barrier for treatment in the lower income population where especially MDIs are deemed to be beneficial. A clinical study [42] observed that the use of computer-guided surgery with a partially guided protocol does not completely compensate the level of the surgeon’s experience. We suggest that during the drilling procedure in the atrophic maxilla, a “bi-digital” palpation of the fragile alveolar process is carried out, with gentle burr repositioning whenever mispositioning is felt, and a slow drilling procedure using the drill as an osteotome. The latter can reduce the drill-to-bone contact with a short frictional force exposure as suggested by Sannino et al. [43]. In Figure 9, there seems to be no difference between anterior and posterior MDIs with regards to the number of perforations. Prevalence of implant positioning-related complications were evaluated in a cross-sectional CBCT study [44] of 1208 standard dental implants in the maxilla. They found a total of 40/1208 (3.3%) sinus–nasal perforations. The clinical impact of implant perforations into the sinus was extensively investigated by Ragucci et al. [45]. They concluded that the overall survival rate of standard diameter implants into the sinus cavity was 95.6%, without statistical differences according to the level of penetration. The clinical and radiological complications were 3.4% and 14.8%, respectively. The most frequent clinical complication was epistaxis and the most radiological complication was thickening of the Schneiderian membrane. These findings suggest minor clinical influence of sinus/nasal perforations. This corresponds with our anamnestic finding at two years. Despite 21% observed sinus/nasal perforations, only two patients reported some moderate rhino-sinusitis complaints. It is also clear that for better primary stability the surgeon opted for bicortical anchorage of the MDIs which may explain the high incidence of sinus/nasal perforations.

Our clinical study has several limitations that should be recognized. Flapless surgery performed by perforating the mucosa with a drill instead of a punch may be less accurate than open-flap or punch techniques. On the other hand, the latter destroys much keratinized tissue, which is preferred for healing and proper oral hygiene measures. Minimal invasive surgery was also required according to the protocol scrutinized in the study. The follow-up period of two years post-implantation is relatively short; longer follow-up is required and ongoing. A control group with guided flapless surgery or with conventional flapped surgery for comparison is lacking because the study was not designed as an RCT. Random allocation of the participants in the study was not possible due to inclusion requirements such as a healthy medical status and narrow bone ridge. Hence a meta-analysis comparing flapless versus conventional flapped dental implant surgery reported on [46] the increased risk for implant failure in flapless surgery, although no significant in terms of postoperative infection or in regard to marginal bone loss. It was concluded that one of the reasons for higher implant failure in the flapless technique is the increased risk of bone fenestrations and perforations. One may suggest that a randomized clinical trial would be a more scientific approach to further explore whether MDIs could stand the test with regular implants, but this would only be valid if one would want to check the predictability of MDIs in the maxilla, in conditions with less resorbed jaws. However, literature is scarce for maxillary overdentures specifically, it is an interesting future step to be taken because there are indications that MDIs yields comparable outcomes for mandibular overdenture retention [5]. The application of cheaper hardware could indeed be beneficial for many patients. It is obvious that it reduces treatment cost and lowers the barrier to undergo the treatment, especially when extensive bone grafting procedures can be avoided. Another drawback lies in the fact that artifacts (backscatter and beam hardening) around the implants on the radiographic image may limit correct assessment especially with thin bone coverage bucally or palatally. Resilience of the denture could also affect the correct transfer from the planned to the executed procedure, due to soft tissue consistency. Furthermore, evaluation of bone quality was not possible due to the inability to measure Hounsfield Units with CBCT. It has been shown that artifacts in CBCT were always present in the proximity of implants made from titanium, irrespective of the implant position in the jaw [47], hence resulting in doubtful outcome for the true assessment of osseointegration. Another main drawback is that the implants initially lost were not radiologically evaluated because they were removed prior to taking the CBCT. One cannot exclude that malpositioning or bone perforations are associated with the encountered failures.

## 5. Conclusions

It can be concluded that despite a compromised population, aforementioned limitations the minimally invasive procedure and the low treatment cost involved, a failure rate of 17% for the maxillary MDI is clinically acceptable. However—even for experienced surgeons—freehand flapless placement yields a high risk for implant perforation, although this does not necessarily lead to severe or long-term complications.

## Figures and Tables

**Table 1:** Mean bone width at the different measured MDI sections.

Bone Width (mm)	Minimum	Maximum	Mean	SD
Crest (1 mm)	0.00	16.68	5.63	2.84
3 mm	0.00	14.02	5.78	2.68
6 mm	0.00	17.71	6.68	2.86
9 mm	0.00	24.37	6.61	4.07

**Table 2:** Number of perforation at different MDI sites.

Location	No Perforation	Perforation	%
Buccal coronal third (BC)	147	23	13%
Buccal middle third (MB)	145	25	15%
Buccal apical third (AB)	139	31	18%
Palatal coronal third (PC)	144	26	15%
Palatal middle third (MP)	154	16	9%
Palatal apical third (AP)	140	30	17%
Apical tip (Apex)	127	43	25%
Nasal/sinus	133	37	21%

## Supplementary Materials

The following are available online at https://www.mdpi.com/2077-0383/9/7/2120/s1.
