# Supplementary material for: Three-Dimensional Radiographic Outcome of Free-Handed Flaplessly Placed Mini Dental Implants in Edentulous Maxillae after 2-Years Function
Source: J Clin Med. 2020 Jul 5;9(7):2120. doi: 10.3390/jcm9072120 (PMC7408764; doi:10.3390/jcm9072120)
Supplement: Supplementary file 1 [file jcm-09-02120-s001.pdf]

Supplementary Materials:

Three-Dimensional Radiographic Outcome of Free-Handed Flaplessly Placed Mini Dental Implants in Edentulous Maxillae after 2-Years Function

Table 6. Rhinosinusitis symptom severity score: RS-SSS

Patient Name:	Date of Birth:
Date:	File No:

Please Score Your Symptoms 0 (none) 1 (mild) 2 (moderate) 3 (severe)

1. Nasal Congestion (blockage)	
2. Clear Nasal Discharge	
3. Discoloured Nasal Discharge	
4. Facial Pain	
5. Sinus Headache	
6. Upper Jaw Toothache	
7. Disturbed Smelling	
8. Cave-like Speech	
9. Sore Throat	
10. Fever (more than 37.8°C)	
11. Foul Breath	
12. Swelling Around Eyes	
13. Cough	
14. Wheezy Chest	
15. Tiredness	

(1–6: major symptoms, 7–15: minor symptoms)

Symptom Key: 0 – no symptom, 1(mild) – some discomfort, 2 (moderate) – discomfort which sometimes interferes with daily activities and sleep, 3 (severe) – discomfort, regularly preventing participation in work, school and other activities.

Office Use

Scoring: To qualify a patient must have at least one major symptom

Score of 1	
Highest Score of 2 or 3	
Highest Score of 4, 5 or 6	
Highest Score of 7–15 divided by 3	
Total	

Key :

1–3.66: Mild
4–8.66 : Moderate
9 or more: Severe

Global Clinical Assessment: ☐ **None** ☐ **Mild** ☐ **Moderate** ☐ **Severe**

a
